# Rise and metabolic roles of *Vibrio* during the fermentation of crab paste

**DOI:** 10.3389/fnut.2023.1092573

**Published:** 2023-02-24

**Authors:** Tian-Han Xiong, Ce Shi, Chang-Kao Mu, Chun-Lin Wang, Yang-Fang Ye

**Affiliations:** ^1^School of Marine Sciences, Ningbo University, Ningbo, China; ^2^Key Laboratory of Aquacultural Biotechnology, Ministry of Education, Ningbo University, Ningbo, China

**Keywords:** crab paste, swimming crab, fermentation, *Vibrio*, bacterial community

## Abstract

Microbial community may systematically promote the development of fermentation process of foods. Traditional fermentation is a spontaneous natural process that determines a unique nutritional characteristic of crab paste of *Portunus trituberculatus*, However, rare information is available regarding the development pattern and metabolic role of bacterial community during the fermentation of crab paste. Here, using a 16S rRNA gene amplicon sequencing technology, we investigated dynamics of bacterial community and its relationship with metabolites during the fermentation of crab paste. The results showed that bacterial community changed dynamically with the fermentation of crab paste which highlighted by consistently decreased α-diversity and overwhelming dominance of *Vibrio* at the later days of fermentation. *Vibrio* had a positive correlation with trimethylamine, hypoxanthine, formate, and alanine while a negative correlation with inosine and adenosine diphosphate. In contrast, most of other bacterial indicators had a reverse correlation with these metabolites. Moreover, *Vibrio* presented an improved function potential in the formation of the significantly increased metabolites. These findings demonstrate that the inexorable rise of *Vibrio* not only drives the indicator OTUs turnover in the bacterial community but also has incriminated the quality of crab paste from fresh to perished.

## 1. Introduction

By giving the distinctive flavor and taste, traditional fermentation process has been conducted on a catalog of seafoods such as fish (1, 2), shrimp (3), and crab (4). On one hand, the physiologically active substances such as vitamins (3), amino acids (5), and organic acids (6) are produced during the fermentation of aquatic products. On the other hand, some undesirable substances such as hypoxanthine (Hx) and trimethylamine (TMA) could be simultaneously formed (7). Hx is the main cause of the bitter taste and unpleasant smell of aquatic products (8, 9) whereas TMA may induce cancer (10) and cardiovascular disease (11) if taken excessively. In this regard, fermented seafood quality is a hotspot for food safety, which play a vital role for human health.

Traditional fermentation of seafoods is a spontaneous natural process in which autolytic enzymes and microbes jointly develop a unique nutritional characteristic (12). Among them, microbial community could be adaptive and metabolically diverse and may systematically promote the development of fermentation process (13). Particularly, some studies have explored the microbial metabolic functions in the production of volatile flavor compounds from shrimp paste (12) and fermented mandarin fish (14). Like Hx from adenosine triphosphate (ATP) (15), and TMA from trimethylamine N-oxide (TMAO) (16, 17). Therefore, comprehensively elucidating how the microbial community changes and its relationship with fermented substances over fermentation is fundamental for the seafood quality.

As a popular fermented aquatic product, crab paste is made by directly mixing fresh meat of the swimming crab, *Portunus trituberculatus*, with seasonings including salt, sugar, monosodium glutamate, and liquor (4). Twenty-eight metabolites such as amino acids, organic acids, and organic bases have been detected in crab paste based on our previous metabolomic work (7). Although some cultural bacteria such as *Staphylococcus*, *Arthrobacter*, and *Sphingobacterium* have been found in crab paste (18–21). Rare information is available regarding the succession of bacterial community in such an evolving, complex mixture of crab paste and its metabolic contribution to fermented substances, which might be important to the quality control of crab paste.

Therefore, in this study, a 16S rRNA gene amplicon sequencing technology was used to analyze the change of bacterial community structure across the fermentation of crab paste. Combining multivariate statistical analysis and PICRUSt2 functional prediction, we aimed to reveal the following: (1) the dynamics of α-diversity and composition of bacterial community with the fermentation of crab paste, (2) the relationships between bacterial taxa and fermented products of crab paste, (3) the functional potential of *Vibrio* in the formation of fermented products.

## 2. Materials and methods

### 2.1. Crab paste processing

Live seven swimming crabs (205.69 ± 17.25 g) were purchased from a crab aquafarm in Ningbo, China and anaesthetized on ice. Each crab was cleaned using tap water and cut into pieces immediately. For crab paste processing, the crab pieces were mixed with 4% sucrose, 2% salt, 1% monosodium glutamate, and 1% white liquor with 40% ethanol and were packaged in an airtight plastic bag for 7 days-fermentation at 4°C. The samples were respectively taken at 1, 3, 5, and 7 days of fermentation and the fresh crab samples were used as control. All samples were stored at −80°C for further analysis.

### 2.2. DNA extraction, 16S rRNA gene amplification, and illumina sequencing

Genomic DNA was extracted from 0.5 g of crab paste for each sample using the FastDNA Spin kit (MP Biomedicals, USA). The concentration and purity of DNA extracts were measured by a NanoDrop ND-1000 spectrophotometer. The V3–V4 region of 16S rRNA gene was amplified using the primer sets 341F (5′-CCTAYGGGRBGCASCAG-3′) and 806R (5′-GGACTACHVGGGTWTCTAAT-3′) with dual barcodes (21). To reduce the bias during amplification, PCR reactions were performed in triplicate for each sample. Following purification, assessment of fragment size, and quantification, PCR amplicons for each sample were aggregated in equimolar ratios and sequenced with the Illumina MiSeq platform (Illumina, USA) for generating paired end reads. Raw sequence data are available in the NCBI Sequence Read Archive under BigProject PRJNA808815.

### 2.3. Sequence processing

Paired-end reads were merged using FLASH (22). The merged sequences were quality filtered and processed using the QIIME2 pipeline (23). Following chimera detection using UCHIME (24), the remaining high-quality sequences without chimeras were sorted into the operational taxonomic units (OTUs) with a cutoff of 97% sequence similarity using UCLUST (25). To obtain the taxonomic information, the sequence with the highest abundance and coverage in each OTU was selected for assignment against the Greengenes database (release 13.8) using PyNAST (26). After removing the sequences which cannot be assigned to bacteria, a total of 3,288,164 clean reads (mean 93,948 reads per sample) were detected in the 35 crab paste samples. To avoid unequal sequencing depth, the OTU table was rarefied at 44,350 reads per sample for further analysis.

### 2.4. Statistical analysis

Shannon index, Richness, and phylogenetic diversity indices were calculated using QIIME. Pielou’s evenness was calculated using the R package “vegan.” Differences in α-diversity indices and bacterial populations were compared using the Kruskal-Wallis test in the “agricolae” package. A non-metric multidimensional scaling (NMDS) and a principal coordinate analysis (PCoA) were jointly used to visualize the differences in bacterial communities of crab paste between each fermentation time point, with three non-parametric multivariate analyses of dissimilarity based on Bray-Curtis distance using the “vegan” package, including MRPP, ANOSIM, and Adonis. To identify indicative bacteria that associated with fermentation time, the indicators at the OTU level were screened using the R “labdsv” package (27). The indicators of dominant bacteria at the OTU level (with average relative abundance > 0.01% in all samples) were screened with a significant difference between groups (*p* < 0.05) and indicator value (Indval) > 0.5 using the R package “labdsv.” Spearman correlations between indicator OTUs and significantly changed metabolites (7) were calculated and visualized using the R “psych” and “pheatmap” packages, respectively.

To infer the functional potential of *Vibrio*, the OTU table of *Vibrio* was used for predicted 16S rRNA gene copy number by PICRUSt2 (Phylogenetic Investigation of Communities by Reconstruction of Observed States, v2.1.0-b) pipeline (28). Here, only genes involved in the metabolism pathways of significantly changed metabolites of crab paste were predicted. Functional annotations of the genes were obtained according to the KEGG database.^1^ Differences in functional genes were compared using the Kruskal-Wallis test in the “agricolae” package. All analyses and plots were completed in R 4.0.4 (R Foundation for Statistical Computing, Vienna, Austria).

## 3. Results

### 3.1. Change of bacterial community during fermentation

After the sequencing of 16S rRNA amplicons, a total of 3,288,164 high-quality sequences from 35 samples were obtained, with an average of 93,948 ± 21,784 reads per sample. Subsequent analysis was performed on a minimum of 44,350 reads per sample after normalization and homogenization.

**FIGURE 1:**
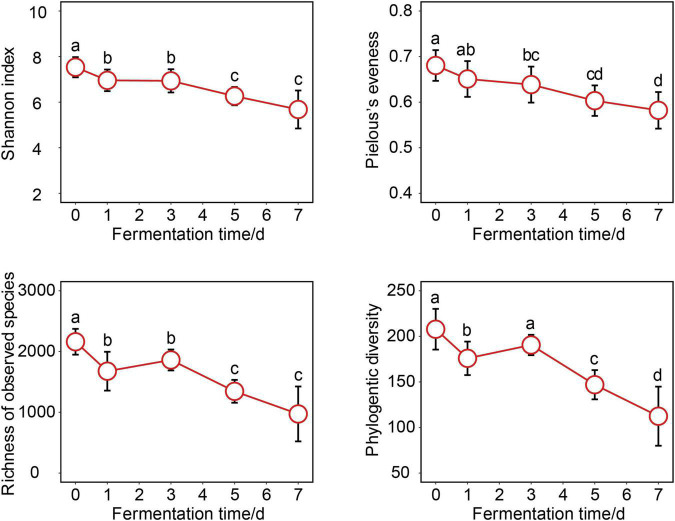
Changes of bacterial α-diversity indices over crab paste fermentation. Data present means ± standard deviation. Different letters indicate significant differences among groups (*p* < 0.05).

To explore change in the bacterial community of crab paste during fermentation, changes of bacterial α-diversity indices were investigated. Bacterial α-diversity of crab paste was at the highest level before fermentation (at day 0), then decreased after fermentation (Figure 1). The Shannon index, richness of observed species, and phylogenetic diversity decreased sharply at the first day of fermentation (*p* < 0.05) while the Pielous’s evenness index showed a significant drop from the third day of fermentation (*p* < 0.05). Although the bacterial α-diversity showed certain fluctuation at day 3, it finally reached the lowest level at the end of fermentation. Overall, bacterial α-diversity indices of crab paste were all reduced over fermentation time.

**FIGURE 2:**
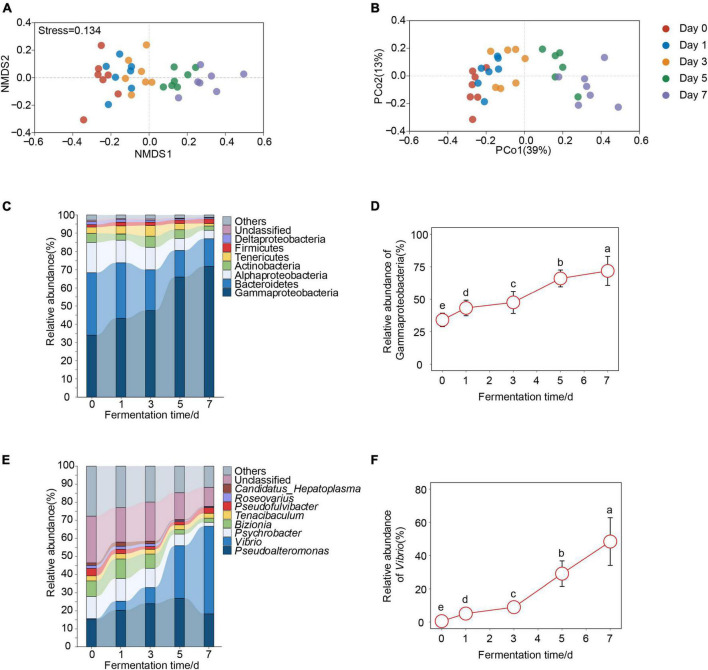
Changes of bacterial communities over crab paste fermentation. Non-metric multidimensional scaling (NMDS) plot **(A)** and principal coordinate analysis (PCoA) plot **(B)** based on Bray-Curtis dissimilarity visualizing compositional variations of bacterial communities of crab paste at day 0 (red), day 1 (blue), day 3 (orange), day 5 (green), and day 7 (purple). The relative abundances of bacterial communities at the phyla/proteobacterial classes **(C)** and genus **(E)** Level in crab paste during fermentation. Species with relative abundance < 1% were classified as “others”. Significantly increased phyla/proteobacterial classes **(D)** and genera **(F)** over crab paste fermentation. Data present means ± standard deviation. Different letters indicate significant differences among groups (*p* < 0.05).

Next, we analyzed whether bacterial community composition of crab paste changed with fermentation time. We found that the bacterial community of crab paste presented a clear succession tendency along the first axis after fermentation if fresh crab samples were excluded (Figures 2A, B). Three non-parametric dissimilarity analyses including MRPP, ANOSIM, and Adonis showed a significant difference between fermentation time points (*p* < 0.05) except between day 0 and day 1 as well as between day 1 and day 3 (Table 1). These observations indicate that the main variation in the crab paste microbiota occurs at the later period of fermentation.

**TABLE 1:** Significance tests of the differences in bacterial communities of crab paste between fermentation time points.

Group	MRPP		ANOSIM		ADONIS	
	**δ**	***p***	***r***	***p***	***F***	***p***
Day 0-Day 1	0.007	0.277	0.062	0.253	1.373	0.190
Day 0-Day 3	0.067	0.003	0.370	0.003	3.005	0.003
Day 0-Day 5	0.227	0.003	0.909	0.004	10.130	0.004
Day 0-Day 7	0.275	0.003	0.983	0.003	13.670	0.003
Day 1-Day 3	0.007	0.277	0.050	0.288	1.271	0.201
Day 1-Day 5	0.175	0.003	0.873	0.003	7.420	0.003
Day 1-Day 7	0.245	0.003	0.983	0.003	11.630	0.003
Day 3-Day 5	0.101	0.003	0.510	0.003	4.234	0.003
Day 3-Day 7	0.216	0.003	0.934	0.003	9.713	0.003
Day 5-Day 7	0.077	0.014	0.381	0.009	3.312	0.016

MRPP, multiple response permutation procedure; ANOSIM, analysis of similarity; Adonis, permutational multivariate analysis of variance.

The changes in bacterial community composition of crab paste over fermentation time were detectable at the phylum/class level (Figure 2C). Seven dominant phyla/classes including Gammaproteobacteria, Bacteroides, Alphaproteobacteria, Actinobacteria, Tenericutes, Firmicutes, and Deltaproteobacteria all changed across time. Of note, the relative abundance of Gammaproteobacteria highly increased over fermentation time (Figure 2D). Among them, the relative abundance of Gammaproteobacteria continued to increase to the extreme dominance (71.9%) at day 7. In contrast, the relative abundances of Bacteroides, Alphaproteobacteria, and Deltaproteobacteria decreased over fermentation time (Supplementary Figure 1A). The bacterial changes in crab paste over fermentation time were also detectable at the genus level (Figure 2E). The average relative abundance of *Vibrio* markedly increased throughout the fermentation, reaching up to 48% at day 7 (Figure 2F). In contrast, the relative abundances of *Psychrobacter*, *Bizionia*, and *Roseovarius* significantly decreased over time (Supplementary Figure 1B). Taken together, these observations indicate a substantial rising of Gammaproteobacteria (mainly *Vibrio*) in the bacterial community of crab paste due to fermentation.

### 3.2. Indicator OTUs associated with fermentation time

**FIGURE 3:**
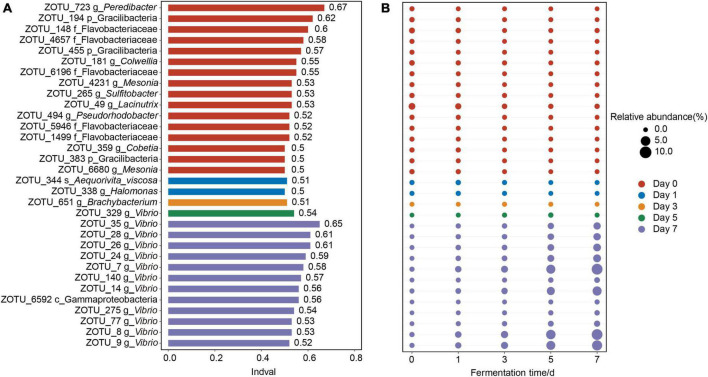
The 32 indicator operational taxonomic units (OTUs) values **(A)** and relative abundances **(B)** of crab paste at day 0 (red), day 1 (blue), day 3 (orange), day 5 (green), and day 7 (purple). The length of the bar in plot **(A)** represents Indval of one indicator OTU. The diameters of the circles in plot **(B)** are proportional to the relative abundances of the OTUs, with the red, blue, orange, green, and purple circles indicating the peak relative abundances at five fermentation time points, respectively.

The bacterial assemblages which characterized the discrete bacterial communities of crab paste due to fermentation were further examined. A total of 32 indicator OTUs were screened out at different fermentation time points (Figure 3). In detail, nearly half of the indicator OTUs were most abundant in the fresh crabs (at day 0), such as five OTUs belonging to Flavobacteriaceae (OTU 148, OTU 4657, OTU 6196, OTU 5946, and OTU 1499), three OTUs belonging to Gracilibacteria (OTU 194, OTU 455, and OTU 383), and two OTUs belonging to *Mesonia* (OTU 4231 and OTU 6680) (Figure 3A). As fermentation proceeded, indicators OTU 344 belonging to *Aequorivita viscosa* and OTU 338 belonging to *Halomonas* were most abundant in the crab paste at day 1 (Figure 3B). Further, only one indicator OTU 651 belonging to *Brachybacterium* presented the highest relative abundance at day 3 and one indicator OTU 329 belonging to *Vibrio* did at day 5 (Figure 3B). At day 7, 11 OTUs (OTU 35, OTU 28, OTU 26, OTU 24, OTU 7, OTU 140, OTU 14, OTU 275, OTU 77, OTU 8, and OTU 9) belonging to *Vibrio* dominated in the bacterial community of crab paste, accompanied by OTU 6592 belonging to Gammaproteobacteria (Figure 3A).

### 3.3. Relationships between indicator OTUs and metabolites

**FIGURE 4:**
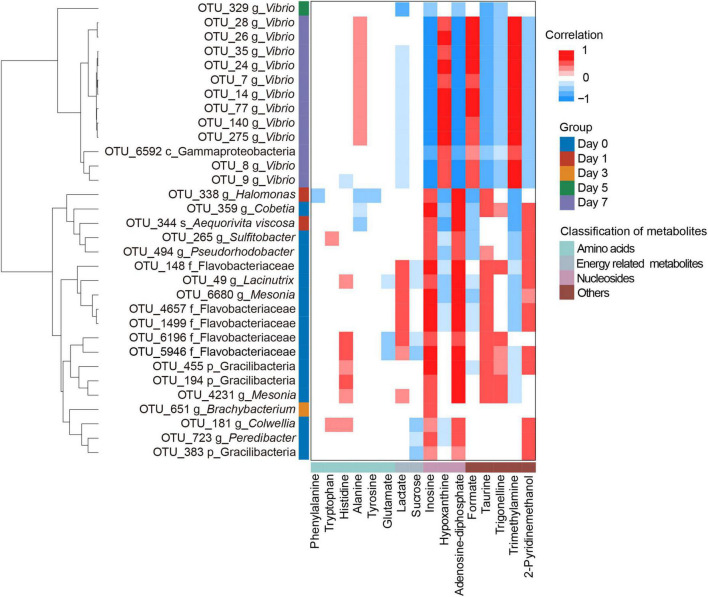
Clustering heatmap showing the Spearman’s correlations between the indicative bacterial operational taxonomic units (OTUs) and the significantly changed metabolites, with red and blue indicating significant positive and negative correlations, respectively.

To understand how bacteria relate to metabolites during crab paste fermentation, we analyzed the relationships between indicator OTUs and significantly changed metabolites based on our previous metabolomic results (7). As expected, the heatmap shows a close correlation between bacteria and metabolites with red denoting positive correlation, whereas blue denoting negative correlation (Figure 4). Notably, almost all of indicator OTUs could be classified into two clusters according to the taxonomic information: cluster I (not *Vibrio*) and cluster II (*Vibrio*) except OTU6592 which could not be assigned to genus due to the limited 16S rRNA gene information. The cluster I was complex during the first three days of fermentation while cluster II tended to be simple and highlighted by one remarkable indicator OTU turnover since the fifth day of fermentation. Specifically, 12 *Vibrio* OTUs overwhelmingly dominated in cluster II with one OTU emerging at day 5. Moreover, the two clusters presented a complementary correlation with metabolites. In detail, most of OTUs in cluster I showed positive correlations with inosine, adenosine diphosphate (ADP), taurine, and 2-pyridinemethanol, whereas negative correlations with formate, Hx, and TMA. In contrast, most of OTUs in cluster II presented reverse correlations with metabolites which highlighted by positive correlations with alanine, formate, Hx, and TMA while negative correlations with lactate, inosine, ADP, taurine, trigonelline, and 2-pyridinemethanol. These observations indicate that the succession of bacterial community may play a vital role in the metabolite formation of crab paste during fermentation.

### 3.4. *Vibrio* function prediction analysis

**FIGURE 5:**
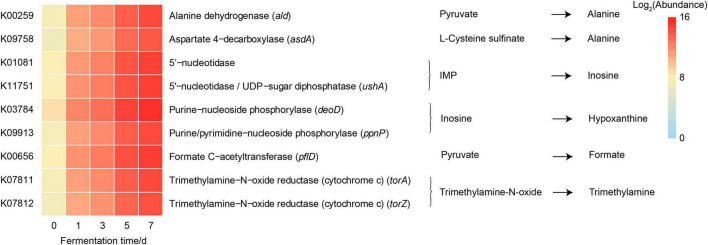
Heatmap showing the abundances of predicted function genes of *Vibrio* involved in the metabolic pathways of significantly changed metabolites of crab paste during fermentation. The data of gene abundances were log2 transformed.

Given the predominance of *Vibrio* in the bacterial community due to fermentation, large differences in functional profiles of the genus *Vibrio* between before and after fermentation were predicted by PICRUSt2. We found a significant increase in the abundances of nine predicted function genes of *Vibrio* (*p* < 0.05) (Table 2 and Figure 5), which were involved in the metabolic pathways of significantly changed metabolites of crab paste during fermentation (7). For example, the abundances of *torA* and *torZ* encoding trimethylamine-N-oxide reductase increased approximately 98-fold at day 7 compared to those at day 0. These results indicate that *Vibrio* may play a critical role in the metabolite changes of crab paste during fermentation.

**TABLE 2:** The abundances of predicted functional genes in *Vibrio*.

KO	Group				
	**Day 0**	**Day 1**	**Day 3**	**Day 5**	**Day 7**
K00259	191.24 ± 68.72^e^	1990.81 ± 813.89^d^	3487.11 ± 1306.30^c^	11221.26 ± 2888.51^b^	18556.90 ± 5444.85^a^
K09758	125.78 ± 46.89^e^	1435.99 ± 608.33^d^	2584.95 ± 947.47^c^	8059.44 ± 2260.10^b^	12935.29 ± 3749.88^a^
K01081	239.46 ± 87.64^e^	2429.31 ± 975.12^d^	4317.18 ± 1607.31^c^	14450.61 ± 3684.46^b^	24546.12 ± 7115.68^a^
K11751	253.56 ± 90.20^e^	2852.81 ± 1216.23^d^	4900.66 ± 1897.43^c^	15194.42 ± 3800.12^b^	24636.19 ± 7247.23^a^
K03784	382.06 ± 137.09^e^	3969.04 ± 1628.83^d^	6943.80 ± 2613.25^c^	22388.37 ± 5764.55^b^	37102.81 ± 10889.60^a^
K09913	189.82 ± 68.53^e^	1987.66 ± 814.45^d^	3487.11 ± 1306.30^c^	11220.97 ± 2888.30^b^	18556.76 ± 5444.73^a^
K00656	268.77 ± 93.20^e^	2932.22 ± 1239.96^d^	5145.58 ± 1956.57^c^	16189.7 ± 4127.10^b^	26295.19 ± 7670.21^a^
K07811	190.24 ± 68.23^e^	1979.95 ± 814.39^d^	3456.54 ± 1307.49^c^	11166.83 ± 2876.03^b^	18546.05 ± 5444.87^a^
K07812	156.75 ± 56.79^e^	1657.57 ± 689.89^d^	2915.83 ± 1110.75^c^	9391.07 ± 2307.23^b^	15433.43 ± 4488.84^a^

Different letters indicate significant differences among groups (*p* < 0.05).

## 4. Discussion

Microorganisms impart crab paste a unique flavor and nutritional composition, but they may also lead to spoilage (18–20). Understanding the dynamic changes in the bacterial community may help in the quality control in seafood fermentation. Our microbial study across 7-day fermentation of crab paste allowed an in-depth look into the dynamics of a developing fermented seafood ecosystem. We observed a consistent decrease in bacterial α-diversity over fermentation (Figure 1). The decrease of bacterial α-diversity due to fermentation has been observed in fermented tempeh (29), kimchi (30), and fish (2). The reason why the great loss of bacterial α-diversity during fermentation may be the overabundance of Gamaproteobacteria which inexorably rising from the fifth day of fermentation (Figures 2C, D). Specifically, merely the relative abundance of Gamaproteobacteria (mainly *Vibrio*) continuously increased with fermentation which accompanied by a substantial decrease in the relative abundances of other phyla. Notably, only *Vibrio* became predominant in the later days of crab paste fermentation (Figures 2E, F, 3A, B), which is consistent with the change characteristics of gut bacteria in the diseased swimming crab (31) and shrimp (32). *Vibrio* comprises about > 100 species (33). some *Vibrio* species such as *V. harveyi*, *V. alginolyticus*, and *V. parahaemolyticus* form pathogenic or symbiotic relationships with marine animals (34–37). This genus seemly had a robust competitive ability and physiological adaption in crab paste environment with high sugar, high salt, and high liquor contents. Such a characteristic change of bacterial community highlighted by an inexorable rise of *Vibrio* may drive the evolution of metabolomic profile of crab paste during fermentation as observed before (7).

Our correlation analysis and functional prediction can confirm this assertion. As we expected, the results of correlation analysis strongly support a vital role of *Vibrio* in the formation of TMA, Hx, formate, and alanine in the crab paste during fermentation (Figure 4). TMA is mainly responsible for the fishy odor and often associated with decomposition of aquatic animals, therefore this simple amine is regarded as well-known fish freshness index (38). An excessive intake of TMA will lead to a TMA poisoning (39) and it has been identified as a uremic toxin (40). TMA has been found to be a product of various types of bacteria such as *Escherichia*, *Shewanella*, *Photobacterium*, and *Vibrio via* the reduction of TMAO (16, 41–44). Of note, in this study, two genes K07811 (*torA*) and K07812 (*torZ*) encoding two isozymes of TMAO reductase (TorA and TorZ) were predicted in the genome of *Vibrio*. TMAO reductase is capable to reduce TMAO to TMA. Moreover, the abundances of these two genes were largely enhanced with the fermentation of crab paste (Figure 5). As such, we speculate that *Vibrio* contributes most to the TMA formation by TMAO reductase system and drives the spoilage of crab paste.

Moreover, *Vibrio* positively correlated to Hx but negatively correlated to ADP and inosine (Figure 4). These three compounds are intermediate metabolites in the pathway of ATP catabolism as observed in the postmortem muscle of the mud crab *Scylla paramamosain* (15, 45). Among them, inosine and Hx are two important determinants of various nucleotide freshness indicators such as *K* value, *Ki* value, and *H* value (46–48). It is generally believed that the breakdown from ATP to inosine monophosphate (IMP) results from autolysis enzyme action, while the further degradation of IMP to inosine and Hx probably results from microbial action (49). In this regard, the production of inosine and Hx may attribute to bacterial metabolism. Moreover, we predicted the genes including K01081 and *ushA* (K11751) which encoding 5′-nucleotidase/UDP-sugar diphosphatase involved in the conversion of IMP to inosine in *Vibrio*. We also predicted the genes including *deoD* (K03784) and *ppnP* (K09913) which encoding purine-nucleoside phosphorylase and purine-pyrimidine-nucleoside phosphorylase involved in the degradation of inosine to Hx in this genus. Notably, the abundances of these four genes were all highly elevated with the fermentation of crab paste (Figure 5). These observations strongly imply the crucial functional potentials of *Vibrio* in the ATP catabolism. Previous evidence supports this notion in which *Vibrio* can secrete extracellular hydrolases decomposing inosine to Hx (50). Given the importance of inosine and Hx in calculating the nucleotide indices, *Vibrio* is further inferred as an important driver in the spoilage of crab paste from the perspective of nucleotide freshness indicator.

In addition, our results suggest that *Vibrio* may contribute to the formate and alanine production in the crab paste (Figures 4, 5). This is in line with earlier observations in which formate and alanine can be produced from glucose and other carbohydrates under the action of *Vibrio* (51, 52). Although formate and alanine are not regarded as the freshness index of seafood, these observations indicate a more diverse metabolic potential of *Vibrio* during the fermentation of crab paste.

Collectively, these findings combined with our previous metabolomic findings demonstrate that the succession of bacterial community highlighted by the rise of *Vibrio* during the fermentation of crab paste could be Darwinism encouraging survival of the fittest by requiring extinction of the unfit. Such a change in microbial ecology indeed correlates with a changed metabolic activity of bacteria, which subsequently driving the quality switch from fresh to spoilt crab paste.

## 5. Conclusion

This study revealed a dynamic change of bacterial community with the fermentation of crab paste which highlighted by consistently decreased α-diversity and overwhelming dominance of *Vibrio* at the later days of fermentation. *Vibrio* had a positive correlation with TMA, Hx, formate, and alanine while a negative correlation with inosine and ADP. In contrast, most of other bacterial indicators had a reverse correlation with these metabolites. Moreover, *Vibrio* presented an improved function potential in the formation of the significantly increased metabolites. Collectively, these findings combined with our previous metabolomic findings demonstrate that the inexorable rise of *Vibrio* not only drives the indicator OTUs turnover in the bacterial community but also is implicated in the quality switch from fresh to spoilt crab paste. In fact, natural microbial fermentation can be a double-edged sword. How to control the microbial fermentation of seafood with great precision merits need to be studied in future.

## Data availability statement

The original contributions presented in this study are publicly available. This data can be found here: https://www.ncbi.nlm.nih.gov/bioproject/PRJNA808815.

## Ethics statement

The animal study was reviewed and approved by Animal Care and Use Committee of Ningbo University.

## Author contributions

T-HX: conceptualization, validation, methodology, data curation, formal analysis, software, and writing—original draft. CS, C-KM, and C-LW: supervision and writing—review and editing. Y-FY: conceptualization, validation, methodology, data curation, formal analysis, funding acquisition, supervision, and writing—review and editing. All authors contributed to the article and approved the submitted version.

## Footnotes

1
http://www.genome.jp/kegg/
