# Supplementary material for: Rise and metabolic roles of Vibrio during the fermentation of crab paste
Source: Front Nutr. 2023 Feb 24;10:1092573. doi: 10.3389/fnut.2023.1092573 (PMC9998518; doi:10.3389/fnut.2023.1092573)
Supplement: Supplementary file 1 [file Table_1.DOCX]

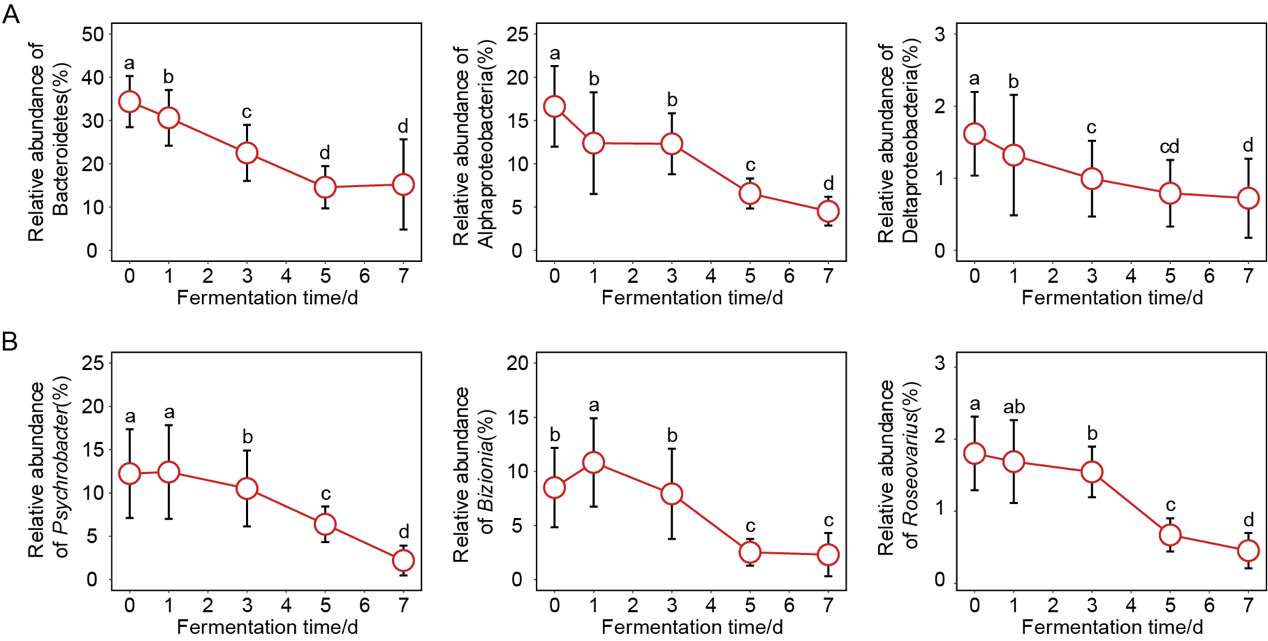


**FIGURE S1** Significantly decreased phyla/proteobacterial classes **(A)** and genera **(B)** over crab paste fermentation. Data present means ± standard deviation. Different letters indicate significant differences among groups (*p* < 0.05).
